# Acknowledgement to Reviewers of *Vaccines* in 2013

**DOI:** 10.3390/vaccines2010179

**Published:** 2014-02-28

**Authors:** Vaccines Editorial Office

**Affiliations:** MDPI AG, Klybeckstrasse 64, CH-4057 Basel, Switzerland

The editors of *Vaccines* would like to express their sincere gratitude to the following reviewers for assessing manuscripts in 2013:

Abel, Kristina
Acedo, L.
Ackerman, Margaret E.
Agadjanyan, Michael
Agwale, Simon M.
Akiyama, Shinichiro
Antony, Paul A.
Barin, Francis
Baron-Epel, Orna
Barry, Peter
Beckett, Charmagne G.
Biacchesi, Stéphane
Bigey, Pascal
Bjorkman, Pamela
Brumme, Zabrina L.
Castro, José María
Chang, Yung-Fu
Chaput, Nathalie
Chiappori, Alberto A.
Cichutek, Klaus
Clayton, Christine
Clements, C. John
Cossarizza, Andrea
Cowan, Anne
De Val, Bernat Pérez
Dean, Gregg
Dempsey, Amanda F.
Derdeyn, Cynthia A.
DiClemente, Ralph J.
Dolstra, Harry
Dory, Daniel
Duerr, Ann
Evans, Claire F.
Fast, Patricia
Field, Mark C.
Flick, Ramon
Forde, Jonathan
Fujihashi, Kohtaro
Fujii, Shin-ichiro
Fujita, Mitsugu
Galli, Laura
Gianella, Sara
Glanz, Jason
Hanley, Sharon J.B.
Harashima, Hideyoshi
Hertz, Tomer
Huh, Jooryung
Inchauspé, Geneviève
Isaguliants, Maria
Iurescia, Sandra
Kim, Kyung-Hyo
Knudsen, Maria L.
Kobinger, Gary
Koblin, Beryl
Koff, Wayne C.
Kortekaas, J.
Lao, T.T.
Lapatra, Scott
Lau, Chuen-Yen
Lavail, Katherine Hart
Law, Barbara
Lichterfeld, Mathias
Lin, Rongtuan
Long, Elisa F.
Magez, S.
Mak, Kwok Kei
Martin, Michael
Marzetta, Carol A.
McCune, Joseph (Mike)
Mcmichael, Andrew J.
Mcshane, Helen
Metzger, David S.
Metzger, Dennis W.
Mitchison, Timothy J.
Mizel, Steven B.
Murtaugh, Michael
Nan, Xiaoli
Nelson, Jennifer Clark
Nemazee, David
Niidome, Takuro
Nowak, Glen
Palmu, Arto A.
Papadimitriuou, Konstantinos
Pedersen, Anders E.
Petrovsky, Nikolai
Preat, Véronique
Purcell, Damian
Pushko, Peter
Rappuoli, Rino
Reisinger, Stephanie J.
Rolland, Morgane
Roxanne, Clark
Rudolph, Carsten
Ruffin, Mack
Schneewind, Olaf
Schuitemaker, Hanneke
Seib, Katherine
Shapiro, Lawrence
Shattock, Robin
Sheets, Rebecca L.
Shih, Mei-chiung
Simard, Edgar
Simon, Raphael
Slack, Mary
Slavcev, Roderick
Smerdou, Christian
Smiley, Stephen
Soren, Karen
Spearman, Paul
Stansbury, James
Stone, Geoffrey W.
Sullender, Wayne
Swamy, Geeta
Tomaras, Georgia D.
Tonyia, Eaves-Pyles
Trottein, François
Tsui, Jennifer
Turner, Stephen
Ueberla, Klaus
Uzonna, Jude
Van Wagoner, Nicholas J.
Villalba, Martin
Vitral, Claudia Lamarca
Wang, Xin
Warfield, Kelly L.
Wicker, S.
Willemen, Y.
Williams, James A.
Wyatt, Richard T.
Yusim, Karina
Zakhartchouk, Alexander
Zhu, Xiaoping

